# Artificial Intelligence and Peer Review: Preserving Integrity in the Pursuit of Efficiency

**DOI:** 10.1590/S1677-5538.IBJU.2025.0538

**Published:** 2025-09-25

**Authors:** José de Bessa, Cristiano Mendes Gomes

**Affiliations:** 1 Universidade Estadual de Feira de Santana Departamento de Urologia Feira de Santana BA Brasil Departamento de Urologia da Universidade Estadual de Feira de Santana – UEFS, Feira de Santana, BA, Brasil; 2 Hospital das Clínicas da Universidade de São Paulo Divisão de Urologia São Paulo SP Brasil Divisão de Urologia, Hospital das Clínicas da Universidade de São Paulo – USP, São Paulo, SP, Brasil

## INTRODUCTION

We aim to contribute to the ongoing discussion about the integration of artificial intelligence (AI) in the peer review process, a topic of increasing relevance in the scientific community.

Large language models (LLMs) are rapidly entering manuscript handling and peer review within scientific publishing. AI tools are most effective in the preliminary stages of review, such as manuscript triage, reviewer matching, and structured integrity checks, while the crucial evaluation of scientific quality remains the responsibility of human reviewers (1–3). Used judiciously and under human supervision, LLMs can help alleviate reviewer shortages and accelerate timelines, particularly in high-volume fields such as medical publishing (1, 2).

However, alongside these efficiency gains come important challenges. LLMs lack the capacity for critical judgment and contextual nuance required in complex scientific evaluation (3–5). Their use also raises concerns regarding transparency, accountability, and the integrity of academic publishing (6, 7). The rapid adoption of AI, progressing faster than regulatory guidance, requires the scientific community to critically assess both its benefits and inherent limitations. Clear policies and responsible disclosure are essential to preserve confidence and maintain rigorous standards in scientific communication (7).

### Evidence and Limitations

Recent pilot studies demonstrate meaningful time savings in early editorial tasks. For instance, the 2024 Fast & Fair peer review pilot at Biology Open reported markedly faster reviewer identification and editorial throughput, with all manuscripts receiving a first decision within seven business days (1). Editors and reviewers noted no decline in review quality but emphasized that the benefits were concentrated in triage and reviewer assignment (2, 3). Similar initiatives confirm that LLMs can reduce manual workload, identifying overlapping as well as additional qualified reviewers (4). Still, their contributions remain confined to early phases and do not replace expert evaluation of methodological soundness, novelty, or validity (5, 6).

### Limits of AI Reviews and Detectors

AI-generated reviews often lack the domain-specific judgment needed to assess unconventional methodologies, subtle flaws, or the broader implications of new findings (4–6). LLMs also struggle with ambiguous data or ethical considerations in trial design—tasks requiring expert intuition beyond learned patterns (5). Detectors for AI-generated text are similarly unreliable, prone to false positives and frequent failures in identifying manipulated or AI-produced content (6, 7). The opacity of both generative AI and detection tools raises further ethical concerns, as their limitations are seldom visible to editors or authors (7).

### Policy Landscape: Transparency and Accountability

Leading organizations have clarified core principles regarding AI in academic publishing. The International Committee of Medical Journal Editors (ICMJE) requires authors to disclose any AI use while remaining fully responsible for accuracy and integrity (8). The Committee on Publication Ethics (COPE) similarly states that AI cannot be credited as an author and stresses transparency and human accountability (8). Together, these positions reinforce a simple principle: AI may assist, but human judgment must prevail.

### Practical Disclosure for Authors

Authors increasingly employ AI for language polishing, reference formatting, and drafting (2, 4). Disclosures should include the tool or model used, access date, and the specific tasks performed (e.g., grammar, figure legend editing). Authors must confirm that they have verified all AI-assisted content and have not uploaded confidential or identifiable information to public systems (8).

### Good Practice for Reviewers

Undisclosed AI use in peer review is increasingly reported (4, 6), often resulting in generic or checklist-driven critiques (5, 7). Some AI-influenced reviews demand standards suited to high-impact generalist journals, disregarding the aims, scope, or audience of specialized publications (7). This mismatch occurs because LLMs optimize for comprehensive standards, not journal-specific context (9). Consequently, reviewers relying on AI without oversight risk producing evaluations misaligned with editorial mission and expectations (6, 7).

Reviewers should disclose whether AI was used and for which steps (8), refrain from uploading confidential manuscripts to public tools (9), and confirm that they evaluated quality in relation to the journal's aims and scope. Checklists such as STROBE and CONSORT remain valuable when applied with context-sensitive, critical oversight (7).

### Key Points for Responsible AI Integration

We propose the following considerations for journals seeking to balance efficiency and integrity in adopting AI-assisted editorial processes:

- Dual disclosure: Authors and reviewers disclose how AI was used, specifying tool/model, access date, and tasks performed (7, 9).
- Allowable vs. prohibited uses: Permitted tasks include triage (scope/fit), language polishing, structured summarization, and checklist assistance (2, 4). Prohibited uses include end-to-end review generation, reliance on AI without human verification, and uploading confidential content to public models (6).
- Detector caution: AI detectors may serve as screening aids but should never be the sole basis for editorial decisions (6, 7).
- Confidentiality and security: Preference should be given to secure, organization-approved AI tools that protect confidentiality and enable audit logs (8, 9). Until institutional solutions are more widely available, policies should encourage best practices without creating inequities.
- Ongoing evaluation: Monitor effects on editorial speed, workload, satisfaction, and error rates, updating policies as evidence accumulates (9).

## CONCLUSIONS

With clear rules, dual disclosure, and safeguards that preserve human oversight, AI can serve as a valuable assistant in peer review—enhancing efficiency without compromising impartiality, scientific rigor, or the trust that underpins scholarly communication (2, 7, 9).

## DISCLOSURES

During the preparation of this manuscript, the authors used several AI-assisted technologies. The LLM ChatGPT-5 (Thinking model, last accessed September 12, 2025) was employed for language refinement, formatting, and structured summarization. Grammarly was used for grammatical review, and SciSpace for bibliographic verification. Following the use of these tools, all content was thoroughly reviewed, edited, and approved by the authors, who take full responsibility for the accuracy and integrity of the published work.

## Data Availability

All data generated or analysed during this study are
